# New measurements of digital technology use: the Immersion in Digital Life and Quality of Digital Experience scales

**DOI:** 10.3389/fpsyt.2025.1595536

**Published:** 2025-06-30

**Authors:** Joanna Witowska, Ruth Ogden, Christine Schoetensack, Katarzyna Goncikowska, Marc Wittmann, Vanda Černohorská, Nuria Codina, Chantal Martin-Soelch, Mónica Fernández Boente, Georgina Giner, José V. Pestana, Julie Papastamatelou

**Affiliations:** ^1^ Institute of Psychology, The Maria Grzegorzewska University, Warsaw, Poland; ^2^ School of Psychology, Liverpool John Moores University, Liverpool, United Kingdom; ^3^ Institute of Psychology, Polish Academy of Sciences, Warsaw, Poland; ^4^ Institute for Frontier Areas of Psychology and Mental Health, Freiburg, Germany; ^5^ Institute of Philosophy, Czech Academy of Sciences, Prague, Czechia; ^6^ Department of Social Psychology and Quantitative Psychology, University of Barcelona, Barcelona, Spain; ^7^ Clinical and Health Psychology Unit, Department of Psychology, University Fribourg, Fribourg, Switzerland

**Keywords:** immersion, digital experience, digital technology, measurements, scale development

## Abstract

**Introduction:**

Current methods of assessing digital technology use fail to adequately capture a holistic picture of how individuals experience digital technology during daily life. This is because current measures focus on measuring the frequency/duration of specific forms of technology use *or* problematic use. This research aimed to create two general measures of digital technology use and experience, respectively, which are flexible amid technological changes.

**Methods:**

The measured constructs were specified via bottom-up analysis of an international qualitative study (N=300) on post-covid digital practices. Across three studies we developed and validated the measures using data from 2227 participants.

**Results:**

Exploratory and Confirmatory Factor Analyses indicated that the Quality of Digital Experience Scale contains 26 items, measuring individuals’ perceptions and overall experience of digital technology usage and its impact on Well-being, Time and Efficiency, and Social Connectedness. The second scale, the Immersion in Digital Life Scale consists of five separate questions concerning individuals’ estimation of how much digital technology is present in different areas of life.

**Discussion:**

The scales offer reliable measurements of individuals’ interactions with technology in the digital era. Their ability to capture engagement beyond frequency and duration will facilitate greater understanding of the complexities of the positive and negative impacts of digital practices on individuals and societies.

## 1 Introduction

The last two decades have seen the rapid proliferation of digital technology (DT) in work, social and personal life (1). As a result, people now live in a state of “permanent connectivity” enabling them to be contacted at anytime, anywhere, and to seek information and stimulation with greater ease and immediacy than ever before (1, 2). Such significant changes in the way in which individuals and societies work, socialize, and interact with each other may have profound impacts on social norms, cognitive functioning, health and well-being (see 3, 4).

To date, a primary focus of global research efforts has been to explore, quantify, and model the impact of DT on a range of psychological, physical, emotional, economic and social outcomes (3, 5). However, at present, our ability to comprehensively measure DT use is impaired by the limited scope of existing measures, which are often (1) specific to an individual platform or device, (2) prioritize the measurement of problematic digital activity, (3) focused on the quantification of DT use rather than the experience of use, and 4) lacking in adequate psychometric validity (6–8).

Most self-report instruments for the assessment of digital activity focus on the measurement of specific forms of DT use, such as the use of the internet (9, 10), information and communication technologies (11, 12), certain digital devices (13) and platforms or apps (14). This is problematic because the rate of technological development far out-paces the creation of scales designed to measure the impact of technologies. As a result, academics are often left researching the impact of current technology use with tools developed and validated for use with outdated devices and platforms and are thus failing to capture the impact of new innovations. One way to overcome this issue is to measure the general and comprehensive construct of “digital technology”, without referencing specific platforms or devices.

When measures do explore digital experience more broadly, they typically focus on specific contexts, for example, the Digital Stressors Scale (15) or the Leisure Internet Usage Scale (16) only assess experiences of DT use in the workplace or leisure time, respectively, and the Mobile Phone Affinity Scale (17) only measures experience of phone use and no other forms of digital media. This specificity is problematic because DT is becoming exponentially embedded into life, and people rarely use a singular digital device. As a result, measures which focus on a singular form of DT fail to capture the totality of digital engagement.

Furthermore, the overwhelming majority of validated instruments are designed to measure or detect problematic or pathological forms of digital activity (13) rather than use and experience when non-problematic or non-pathological. This is in part because measures are focused on the frequency or duration of digital use, or the impact of digital deprivation, rather than the measurement of digital experience itself. As a result, a number of studies focus on the negative impact of media and DT on psychological well-being (e.g. 18, 19) while understanding of non-problematic DT use, and factors associated with digital flourishing are often poorly understood (20).

Whilst existing measures are effective for measuring specific forms of DT use, in specific circumstances, their specificity prevents them from capturing a full picture of an individual’s use and experience of DT in general, even in problematic/pathological users. Given that DT has spread to almost every aspect of life (21), measures of DT which reflect the extent to which it is embedded and experienced in different elements of life are required. To enable future research to obtain a more holistic picture of an individual’s digital engagement and their experiences of DT, new measurement tools are required which capture usage and experiences of digital daily life.

The current paper therefore presents the validation of two measures of digital engagement. The first scale measures quality of digital experiences during daily life (Quality of Digital Experience Scale; QDES), while the second one assesses the extent to which individuals are immersed in DT (Immersion in Digital Life Scale; IDLS). The QDES was designed to assess respondents’ experiences of *Well-being*, *Social Connectedness*, and *Time and Efficiency* when using DT. The IDLS was designed to measure the extent to which different elements of life were completed digitally (e.g. communications, free time, social life). For the purposes of this study, well-being was conceptualized through the lens of positive psychology, specifically in alignment with Seligman’s (22) PERMA model. The PERMA model conceptualizes well-being as a multifaceted construct involving positive emotions, engagement, relationships, meaning, and accomplishment. This model proposes that flourishing and overall well-being and satisfaction with life can be increased, and distress can be decreased, by focusing on and enhancing these areas of life. Although the content of the QDES and IDLS were inductively derived from semi-structured interviews, the final subscales of the QDES align with Seligman’s conceptualization of well-being.

Unlike existing measures, these new scales are 1) independent of technological changes, i.e. they do not refer to a specific platform, tool, DT device or specific online behavior (e.g., only gaming or online shopping); 2) assess individuals’ immersion in the digital world and the quality of their digital experiences in a neutral way, i.e. they do not focus on pathological usage and do not simply measure frequency or duration of use; 3) gauge the quality of individuals’ experience of using DT by exploring positive outcomes as well as negative ones; 4) are not limited to a specific environment e.g. work or personal life, instead measuring digital engagement across daily life.

## 2 Overview of the studies

**Figure 1:**
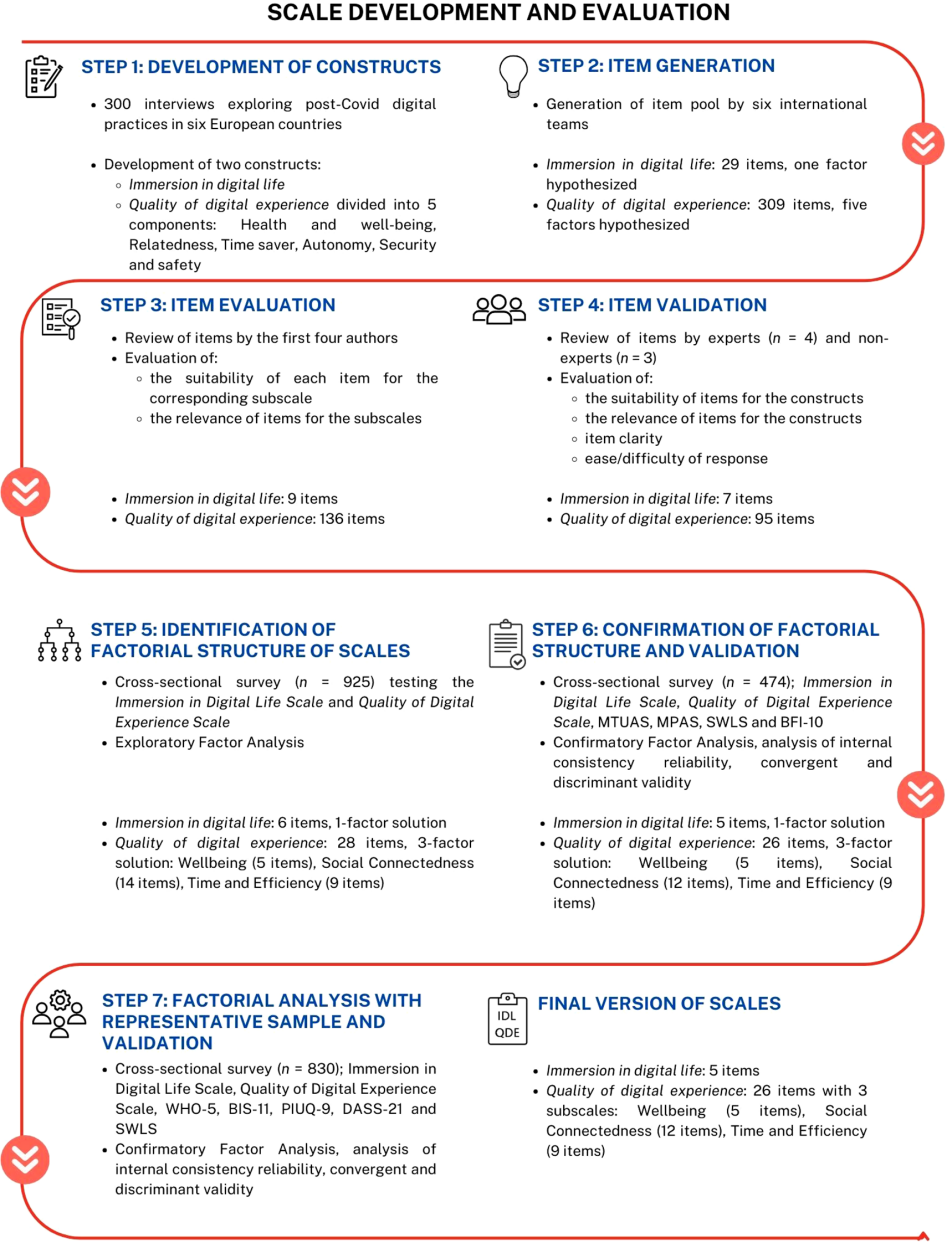
Schematic overview of questionnaire development.

The QDES and IDLS were developed using a mixed methods approach in which interview data was analyzed to develop initial questionnaire items (study 1), which were then tested in studies 2 and 3. A schematic of this process is presented in **Figure 1**.

## 3 Study 1. Scale development and initial evaluation

Items in the QDES and IDLS were generated bottom-up from the thematic analysis of 300 semi-structured interviews with people in the UK, Poland, Germany, Switzerland, Czech Republic and Spain, exploring people’s everyday use and experience of DT.

Based on these interviews, a definition of DT was developed: “*devices, systems, services, data or processes that use digital information in some way, for example, computers, tablets, mobile phones or smart TVs, among other devices. These technologies may be used for various purposes such as communication, entertainment, work, education, and everyday tasks*.”

Analysis of the interviews revealed that participants associated DT use, most of all, with social relationships and communication with friends and family, managing daily tasks and time, as well as relaxation and leisure. Critically however, the degree of pervasiveness and immersion in DT in these life domains varied between participants. We therefore sought to create a simple self-report measure of how much individuals engaged with DT in the above mentioned activities of everyday life.

Analysis of participants’ experiences of using DT revealed five main themes associated with DT use: health and well-being, relatedness, time saving, autonomy, safety and security. These themes were initially defined as potential factors leading to the experience of DT use.

*Health and well-being* – the usage of DT in a manner which positively or negatively impacts physical health including sleep, physical activity, rest and psychological functioning, e.g., feelings, self-esteem, concentration (vs. intrusion, distraction) and memory.

*Relatedness* – the usage of DT in a manner which supports experiencing warmth, bonding, and care, connecting to and feeling significant to others, social support and inclusion.

*Time saving –* the usage of DT in a manner which saves time in life (including ease, flexibility, speed, efficiency).

*Autonomy* - the belief that individuals choose their own behaviors and actions connected to DT and that they have control over it.

*Security & safety* - the usage of DT in a manner which enhances individual safety and security in digital life.

These themes align with existing concepts in psychology referring to quality of life (e.g. 23) basic psychological needs (24) and Seligman’s (22) theory of human flourishing including five fundamental aspects of a good life: positive emotions, engagement, relationships, meaning, and accomplishment. They also highlight the main questions in cyberpsychology regarding advantages and disadvantages of DT and its impact on health, well-being and social relations (3), the discussion on autonomy in DT usage and the right to disconnect from work (see 25) as well as the growing interest in safety in cyberspace (26).

### 3.1 Item development and selection

The pool of items was created by six teams from six European countries based on their interviews and referring to the above-mentioned facets of DT use. Twenty-nine items, which referred to the presence of DT in everyday life, were generated and a total of 309 items in five factors which build experiences of DT use. See **Supplementary Material**: *Section 1.1: Study 1 Details of item development and selection* for further details on the process.

### 3.2 Method

#### 3.2.1 Participants

952 individuals participated. Participants were eligible to take part if they resided in the UK, were aged 18 or above and spoke English fluently. Participants were recruited from Prolific.co using opportunistic sampling. 27 participants were excluded because 1) they did not meet the eligibility criteria (N=1), 2) completed the questionnaire more than once (N=2), 3) had a survey completion time > 3*SD*s above the mean (N=1), or 4) failed either of the two attention check questions (N = 23), leaving a final sample of 925 participants. Demographic details of participants are included in *Section 1.2: Study 1 Demographic details of study 1 participants* in the **Supplementary Material**.

#### 3.2.2 Procedure

Ethical approval was obtained from Liverpool John Moores University Research Ethics Committee (Approval reference number: 23/PSY/041). Data collection followed the principles of the Declaration of Helsinki and was initiated and completed on June 20th, 2023. Participants were compensated £9 pro-rata for participation. All participants gave informed electronic consent. Mean study completion time was 10.48 minutes.

#### 3.2.3 Materials

*Demographics:* Participants provided their gender, age and highest level of education.

The *Quality of Digital Experience Scale* (QDES) consists of 95 items referring to quality of DT usage in specific aspects of life. Participants respond to questions using a 5-point Likert scale with answers from *strongly disagree* to *strongly agree*.

The *Immersion in Digital Life Scale* (IDLS) consists of 7 questions referring to the presence of DT in individuals’ everyday life. Participants respond to questions using a Visual Analogue Scale which ranges from 0 (*not at all digital*) to 100 (*completely digital*).

### 3.3 Analytic strategy

To test the structure of the QDES, Exploratory Factor Analysis (EFA; Principal Factoring Axis, Oblimin rotation) was conducted using SPSS (version 28). Bartlett’s test of sphericity and the Kaiser-Meyer-Olkin (KMO) measure of sampling adequacy revealed perfect feasibility of data for EFA (27), with statistically significant values for Bartlett’s test of sphericity, and KMO =.963.

The analytic strategy included 1) eliminating potential items with loadings lower than .40 (28) and 2) keeping the measure relatively short and easy to complete with strong reliability. We therefore sought to eliminate items that may meet the loading cut-off, but their elimination did not change the scale’s reliability, and we sought to avoid reversed items where possible.

The same requirements were adopted for the IDLS. We conducted EFA with a forced one-factor solution, which showed that seven questions built a one-factor model with perfect feasibility of data with statistically significant values for Bartlett’s test of sphericity, and KMO =.77. The full exploratory method suggested two factors which were not interpretable at all, so we followed our theoretical assumptions regarding the scale that implied a one-factor model. Internal consistency was assessed with Cronbach’s alpha where the threshold of acceptability was .70 (29).

### 3.4 Results

#### 3.4.1 Factor structure of the Quality of Digital Experience Scale

The EFA identified 13 factors, however 10 of these were uninterpretable and did not meet any theoretical or psychometric assumptions. Three clear factors were, however, identified by the scree plot, explained variance, and pattern matrix: these were Well-being, Social Connectedness and Time and Efficiency.

Factor 1: Well-being.

The well-being factor describes the influence of DT use on well-being. It excluded items referring to physical health which did not meet criteria. It initially included 6 items with loadings ranging from .44 to .63. One item was removed (*When I use digital technology, I feel relaxed*) because of a very strong similarity to another item (*Using digital technology helps me to relax*) in wording and meaning. The removed item yielded a lower loading (.47). The final subscale consisted of 5 items with strong reliability *α* **= .**84, explaining 24.7% of variance.

Factor 2: Social Connectedness.

In line with the assumptions from study 1, this factor refers to themes of relatedness, specifically the role of DT use in maintaining and supporting social relationships as well as facilitating bonding and belonging. Initially, 15-items built this factor; however, one item with the lowest loading was removed. The remaining 14 items showed loadings ranging from .54 to .82 with strong reliability, *α* = .94, explaining 11.8% of variance.

Factor 3: Time and Efficiency.

The *Time and Efficiency* factor was in line with themes identified in Study 1. It describes the use of DT as a way to save time and promote efficiency in everyday life. The initial factor consisted of 13 items; the four lowest loading items were removed. These included two reversed items, and one item that was similar to a higher loading item. Their removal led to greater reliability of the subscale with the nine remaining items loading from .54 to .79 with strong reliability, *α* = .92 explaining 4.4% of variance.

#### 3.4.2 Structure of the Immersion in Digital Life Scale

The EFA resulted in the removal of one item, which had the lowest loading (.41). This step improved the reliability of our scale. The final scale contained six questions with loadings from .61 to .75 explaining 46.4% of variance with strong reliability *α* = .76. The results show that Immersion in Digital Life can be measured as a unified construct using the mean score of all the questions.

The conducted analyses resulted in two scales for further testing: The QDES with three subscales consists of 28 items and the IDLS consists of six questions.

## 4 Study 2: Confirmation of the factor structure and validation of the scales

Study 2 aimed to confirm the structure of the scales and their construct and concurrent validity. To test the concurrent validity (convergent and discriminant) we selected psychological constructs relevant for cyberpsychology research (e.g. 3) and theoretically close to the topic of the scales (convergent) as well as constructs that did not seem to be related to the scales (divergent).

We explored the associations between our scales and other aspects of DT measured by *The Mobile Phone Affinity Scale* (MPAS; 17) and *The Media and Technology Usage and Attitudes Scale* (MTUAS; 30). The MPAS was considered to be a suitable measure for our study for two reasons: (1) The construct measured (relationship with the mobile phone) is conceptually similar to the quality of digital experiences assessed by our novel instrument despite the fact that the scope of the MPAS is narrower due to its exclusive focus on mobile phones rather than more general DT use. (2) The MPAS showed very good internal consistency, its subscales good concurrent validity and high loadings of individual items, as assessed in a confirmatory factor analysis conducted by Bock et al. (17). The MTUAS (30) represents one of the few validated measures of DT use that assesses many different types of digital engagement. We selected this instrument due to its comprehensiveness, i.e. its ability to capture both use and attitudes towards DT, the former of which was expected to relate to the IDLS while the latter was anticipated to correlate with the QDES.

We also examined relationships between constructs measured by our scales and the *Satisfaction with Life Scale* (31), hypothesizing that the IDLS would be negatively correlated with satisfaction with life, whereas the QDES would be positively correlated. Given existing evidence that personality traits are associated with aspects of digital life (32–34) we also measured personality traits (35).

### 4.1 Method

#### 4.1.1 Participants

Participant eligibility criteria, the sampling method and exclusion criteria were identical to those used in Study 1. 501 participants completed the survey; however, 27 were excluded (N=1: no UK residency; N=26: reasons mentioned in Study 1) resulting in a final sample of 474 participants. Demographic characteristics of participants are displayed in *Section 2.1 Demographic details of study 2 participants* in the **Supplementary Material**.

#### 4.1.2 Procedure

Liverpool John Moores University Research Ethics Committee approved our study prior to data collection (23/PSY/041). The procedure was identical to that of Study 1. Data collection was started and completed on July 4^th^, 2023. Mean survey completion time was 13.12 minutes.

#### 4.1.3 Materials

Participants answered questions about their gender, age and highest level of education, the QDES (28 items) and the IDLS (6 items). They also completed versions of 1) The Media and Technology Usage and Attitudes Scale (MTUAS; 30), which measured the use of and attitudes towards DT. 2) The Mobile Phone Affinity Scale (MPAS; 17), which assessed DT use. 3) The Satisfaction with Life Scale (SWLS) (31) as a measure of contentment with life. 4) The Big Five Inventory (BFI-10; 36) measured personality by assessing levels of five traits: extraversion, agreeableness, conscientiousness, neuroticism, and openness. Further details on these scales can be found in the **Supplementary Material** (*Section 2.2: Study 2 materials*) and the reliability of these scales is presented in *Section 2.3 Descriptive statistics and reliabilities for all the variables included in Study 2.*

### 4.2 Analytic strategy

#### 4.2.1 Quality of Digital Experience Scale

Confirmatory Factor Analysis (CFA, maximum likelihood) of a three-factor model using IBM SPSS AMOS 28 was conducted to confirm the structure of the QDES. In accordance with widely used statistical guidelines, we report the following fit indices for each model: the model chi-square, degrees of freedom (*x2/df*), RMSEA, CFI and SRMR (37). However, we prioritized the CFI and SRMR because the RMSEA index is not particularly suitable for models with less than 25 degrees of freedom (see 38) and the large sample size renders chi-squared liable to type 1 errors (39). The following criteria for the model fit indicators were used: *x2/df <*5; RMSEA < 0.08, CFI > 0.90 and SRMR < 0.08 (40, 41). Although the widely accepted loading of items is >.40, we decided to take a more conservative approach aiming for loadings close to .70.

Internal consistency was assessed with Cronbach’s alpha where the threshold of acceptability was .70 (29) and additionally with composite reliability (42); the split-half method with even-odd items comparison was additionally conducted. The Guttmann split-half coefficient, correlation coefficient (between forms) and Spearman-Brown formula were calculated. The higher the value the better the reliability with an accepted value of at least .70.

#### 4.2.2 Immersion in Digital Life Scale

CFA of a one-factor model using IBM SPSS AMOS 28 was conducted to confirm the structure of the IDLS. The same criteria were used for testing this scale except in the case of item loadings: here we followed a less conservative approach and accepted values of >.40 in line with common practice. The reason behind this is the low number of tested items.

### 4.3 Results

#### 4.3.1 Quality of Digital Experience Scale

The conducted CFA showed that the χ2 for the model was significant (*χ2 = *717.633; *df* = 292; *p* < 0.001). The model showed a good fit CMIN/DF = 2.888; RMSEA = .063; CFI = 0.926; SRMR = 0.0542. We aimed to have a concise scale that would be easy to complete for participants, without compromising psychometric properties and therefore made some step-by-step modifications in the questionnaire based on the loadings of the items. Two items were removed from the subscale *Social Connectedness* (loadings <.70) with no effect on content validity. Based on the modification indices, we identified that adding four covariances improved the model fit. The final model therefore had an excellent goodness of fit with the following indicators: CMIN/DF = 2.458; RMSEA = 0.056; CFI = 0.948; SRMR = 0.049. All items revealed loadings higher than .70.

The final *Social Connectedness* subscale therefore consisted of 12 items, *Time & Efficiency*: 9 items, *Well-being*: 5 items. **Supplementary Figure S1** in the **Supplementary Material** presents the final 26-item model of the QDES.

#### 4.3.2 Immersion in Digital Life Scale

The CFA showed that the *χ2* for the model was significant (*χ2 = *15.918; *df* = 5; *p* < 0.007). The model did not show acceptable indicators of goodness of fit CMIN/DF = 3.184; RMSEA = .22; CFI = 0.75; SRMR = 0.098. To improve the model fit, one covariance was added (CMIN/DF = 2.445; RMSEA = .055; CFI = 0.99; SRMR = 0.038), but one of the items revealed a low loading (.40). Removing this item produced a better goodness of fit (CMIN/DF = 3.184; RMSEA = .068; CFI = 0.98; SRMR = 0.0332). EFA supported the decision to remove this item. Without including item no. 2, the results showed a clear one-factor structure. However, when item 2 was included, the analysis indicated the presence of two factors in the exploratory model. The theory aligns with the unified construct. **Supplementary Figure S2** in the **Supplementary Material** shows the final 5-item model of the IDLS.

#### 4.3.3 Internal consistency reliability

The internal consistency of the QDES and each subscale shows excellent reliability: Well-being: *α* = .87, *CR* = .86; Social Connectedness: *α* = .95, *CR* = .94; Time and Efficiency: *α* = .92, *CR* = .92; total score of the QDES: *α* = .94, *CR* = .99. The internal consistency of the IDLS presents good reliability as well: *α* = .74, *CR* = .76. Spearman-Brown, Guttman and the correlation coefficient are good for both scales (see *Section 2.5: Spearman-Brown, Guttman and correlation coefficient obtained in Study 2 and Study 3* in the **Supplementary Material**).

#### 4.3.4 Convergent and discriminant validity

The descriptive statistics of all the variables are presented in *Section 2.3 Descriptive statistics and reliabilities for all the variables included in Study 2* and *Section 2.4 Descriptive statistics for Immersion in Digital Life and Quality of Digital Experience in Study 2 and Study 3* in the **Supplementary Material**.

**Table 1** presents the intercorrelations between the different dimensions of the QDES and IDLS. The strongest correlations were found between overall IDLS and its components. IDLS: Free Time correlated with overall IDLS and QDES: Well-being. Overall QDES and its subscales were strongly correlated, with QDES: Time and Efficiency moderately correlated with Well-being and Social Connectedness, which were also strongly linked to each other.

**Table 1:** Study 2: Intercorrelations between the Immersion in Digital Life (IDL) domains (1 to 5) and the overall Immersion score as well as the Quality of Digital Experience (QDE) subscales (7-9) and the overall Quality of Digital Experience score.

	IDL: SR	IDL: CwFam	IDL: CwFriends	IDL: Freetime	IDL: Managing time	IDL	Time & efficiency	Wellbeing	Social connect.	QDE
IDL: Social Relationships	–									
IDL: Communication with family	.42**	–								
IDL: Communication with friends	.67**	.42**	–							
IDL: Free time	.34**	.27**	.32**	–						
IDL: Managing time	.34**	.28**	.36**	.31**	–					
Overall IDL score	.76**	.65**	.73**	.69**	.65**	–				
QDE: Time and efficiency	.17**	.10**	.12*	.22**	.34**	.29**	–			
QDE: Well-being	.26**	.16**	.18**	.41**	.22**	.39**	.38**	–		
QDE: Social connectedness	.40**	.21**	.34**	.24**	.26**	.41**	.36**	.64**	–	
Overall QDE score	.36**	.20**	.29**	.33**	.34**	.45**	.69**	.78**	.90**	–

IDL: SR=IDL: Social Relationships; IDL: CwFam=IDL: Communication with family; IDL: CwFriends=IDL: Communication with friends; SocialConnect.=QDE: Social connectedness. *p <.05; **p <.01.

**Table 2** shows the correlations between different digital behaviors and psychological attributes with various dimensions of the QDES and IDLS.

**Table 2:** Study 2: Intercorrelations between the Immersion in Digital Life (IDL) domains, the overall Immersion score and the subscales and overall score of Quality of Digital Experience (QDE) with different behaviors and trait measures.

	IDL: SR	IDL: CwFam	IDL: CwFriends	IDL: Free time	IDL: Managing time	IDL	Time & efficiency	Well-being	Social connect.	QDE
MTUAS: Text messaging	.22**	.14**	.23**	-.01	.21**	.20**	.17**	-.01	.25**	.22**
MTUAS: Smartphone usage	.26**	.22**	.24**	.09*	.28**	.29**	.21**	.05	.27**	.25**
MTUAS: Social media usage	.28**	.12**	.27**	.11*	.15**	.24**	.12*	.05	.26**	.21**
MTUAS: Internet searching	.18**	.06	.21**	.22**	.24**	.26**	.16**	.12*	.14**	.18**
MTUAS: Media sharing	.14**	.12**	.13**	.21**	.20**	.22**	.07	.11*	.14**	.14**
MTUAS: Phone calling	.00	.01	-.03.	-.03	.10*	-.01	.10*	.02	.14**	.13**
MTUAS: TV watching	.10*	-.01	.05	-.01	.06	.04	.09	.08	.14**	.14**
MTUAS: Positive attitude	.24**	.15**	.20**	.29**	.28**	.35**	.59**	.48**	.45**	.61**
MTUAS: Negative attitude	-.05	-.01	.01	-.05	-.10**	-.07	-.32**	-.35**	-.35**	-.42**
MTUAS: Anxiety/ dependency	.20**	.14**	.20**	.21**	.24**	.29**	.22**	.21**	.28**	.31**
MTUAS: Task switching	.06	.03	.07	-.05	.08	.04	-.00	-.05	.05	.02
MPAS: Connectedness	.38**	.26**	.36**	.19**	.34**	.42**	.37**	.35**	.60*	.59**
MPAS: Productivity	.24**	.13**	.25**	.12*	.36**	.30**	.39**	.20**	.43**	.46**
MPAS: Empowerment	.22**	.13**	.21**	.13**	.24**	.27**	.27**	.29**	.46**	.45**
MPAS: Anxious attachment	.25**	.17**	.26**	.16**	.26**	.31**	.22**	.23**	.38**	.37**
MPAS: Addiction	.27**	.19**	.29**	.15**	.21**	.30**	.10*	-.10	.22**	.17**
MPAS: MPAS: Continuous use	.35**	.21**	.33**	.23**	.30**	.40**	.26**	.26**	.40**	.41**
Satisfaction with life	-.05	.01	-.07	-.18**	-.05	-.10*	.06	.03	.16**	.13**
Extraversion	-.01	.01	-.06	-.16**	.04	-.07	-.09	-.06	.07	-.01
Agreeableness	.01	-.03	-.03	-.07	.01	-.03	.08	.04	.13**	.12*
Conscientiousness	-.12**	-.12	-.19**	-.24**	-.14**	-.24**	.05	-.07	-.06	-.03
Neuroticism	.09	.09	.07	.14**	.06	.13**	-.07	-.01	.05	-.00
Openness	.03	-.00	-.00	-.01	.10*	.04	-.07	-.00	-.01	-.04

IDL, SR = IDL, Social Relationships; IDL, CwFam = IDL, Communication with family; IDL, CwFriends = IDL, Communication with friends; Social Connect. = QDE, Social connectedness; *p <.05; **p <.0.

All the subscales and overall scores of IDLS and QDES indicated positive correlations with neutral, positive and negative aspects of the relationship with the mobile phone as measured by the MPAS. QDES: Social Connectedness and overall QDES showed weak positive correlations with all Media and Technology Usage subscales. All the subscales of IDLS and QDES were positively correlated with the positive attitude toward technology scale with the strongest associations for overall QDES and QDES: Time and Efficiency. The correlations for the negative attitudes toward technology scale were negative and significant for overall QDES and its dimensions were weakly linked to anxiety about technology. Some correlations with personality and satisfaction with life were also identified.

## 5 Study 3: Confirming the factor structure on a representative sample and relationships with other constructs

The third study aimed to replicate the structure of the QDES and IDLS on a sample representative of the UK population in terms of age and gender, and to test congruent and discriminant validity using measures that differ from those in Study 2.

The following constructs were used to further validate our measures: Problematic internet use, well-being, satisfaction with life, depression, anxiety, stress and impulsivity. Considering the results of our previous study we aimed to replicate findings regarding satisfaction with life and explore possible associations with other aspects of mental health by adding measures of well-being, depression, anxiety and stress. As in Study 2, we expected that some dimensions of the IDLS would be negatively correlated with well-being and satisfaction with life, and possibly positively connected with negative outcomes. We hypothesized positive correlations between quality of digital experiences and well-being.

The personality factor of impulsivity was assessed as this trait can be defined as a pattern of actions without regard for potentially negative consequences that might follow. In our context, impulsive behavior may be connected to higher immersion in digital life and potentially lower levels of quality of digital experience.

### 5.1 Method

#### 5.1.1 Participants

Participant inclusion criteria matched those of Studies 1 and 2. A sample representative of the UK population in terms of gender and age was recruited by Qualtrics Panels. 1344 participants completed the study, *N*=117 were excluded due to incomplete responses, 17 were removed as the recorded survey completion time was greater than 3 *SD*s above the mean and 380 had incorrectly answered either of two attention check questions. The final sample size was therefore 830 participants (see *Section 3.1 Demographic details of study 3 participants* in the **Supplementary Material** for demographic details).

#### 5.1.2 Procedure

Following the ethical approval by the Liverpool John Moores University Research Ethics Committee (23/PSY/061), data collection was undertaken between October 16^th^ and 27^th^, 2023. The electronic consent process proceeded exactly as in Study 1 and 2. The mean duration of the survey amounted to 11.91 minutes.

#### 5.1.3 Materials

Demographic information was collected as in Studies 1 and 2.

The five-item version of the IDLS and the 26-item version of the QDES were used (same as in Study 2). Participants also completed 1) The Well-being Index (WHO-5; 43) to measure subjective well-being. 2) The Barratt Impulsiveness Scale 11 (BIS-11; 44) to measure trait impulsivity. 3) The 9-item Problematic Internet Use Questionnaire (PIUQ-9; 45) to measure problematic internet use. 4) The DASS-21 (46, 47) to measure depression, anxiety and stress. 5) The Satisfaction With Life Scale (31).

Further details of these measures can be found in the **Supplementary Material** (*Section 3.2: Study 3 materials*) and their reliability is presented in *Section 3.3 Descriptive statistics and reliability study 3*.

### 5.2 Analytic strategy

The requirements regarding factorial analyses remained as in Study 2.

### 5.3 Results

#### 5.3.1 Quality of Digital Experience Scale

CFA showed that the *χ2* for the model was significant (*χ2 = *937.931; *df* = 290; *p* < 0.001). The first model showed an acceptable goodness of fit (CMIN/DF = 4.205; RMSEA = .062; CFI = 0.94; SRMR = 0.047). However, we decided to check modification indices and in total we added five covariances resulting in an excellent fit CMIN/DF = 3.338; RMSEA = .053; CFI = 0.96; SRMR = 0.042 with all items loading higher than .70. **Supplementary Figure S3** in the **Supplementary Material** presents the final model with all the loadings.

#### 5.3.2 Immersion in Digital Life Scale

A one-factor model with five items was tested using CFA. This CFA showed that the *χ2* for the model was significant (*χ2 = *5.811; *df* = 4; *p* = 0.214). The model showed an excellent fit CMIN/DF = 1.453; RMSEA = .023; CFI = 0.99; SRMR = 0.010 with items loading between .60 and.81. **Supplementary Figure S4** in the **Supplementary Material** presents the final model.

#### 5.3.3 Internal consistency reliability

The internal consistency of the QDES and each subscale shows excellent reliability. These are the values: Well-being: *α* = .89, *CR* = .89; Social Connectedness: *α* = .96, *CR* = .96; Time and Efficiency: *α* = .92, *CR* = .92; overall QDES: *α* = .96, *CR* = .97. The internal consistency of the IDLS presents good reliability as well: *α* = .86, *CR* = .83. Spearman-Brown, Guttman and the correlation coefficient for both scales are good (presented in *Section 2.5: Spearman-Brown, Guttman and correlation coefficient obtained in Study 2 and Study 3* in the **Supplementary Material**).

#### 5.3.4 Convergent and discriminant validity

The descriptive statistics of all the variables are presented in *Section 2.4 Descriptive statistics for Immersion in Digital Life and Quality of Digital Experience in Study 2 and Study 3* and *Section 3.3 Descriptive statistics and reliability study 3* in the **Supplementary Material**.

**Table 3** presents the intercorrelations between the different dimensions of the IDLS and QDES. The observed pattern aligns with that found in Study 2, albeit with stronger correlations. For details, please see **Table 1** and **Table 3**.

**Table 3:** Study 3: Intercorrelations between the Immersion in Digital Life (IDL) domains (1 to 5) and the overall Immersion score as well as the Quality of Digital Experience (QDE) subscales (7-9) and the overall Quality of Digital Experience score.

	IDL: SR	IDL: CwFam	IDL: CwFriends	IDL: Free time	IDL: Managing time	IDL	Time & efficiency	Well-being	Social connect.	QDE
IDL: Social Relationships	–									
IDL: Communication with family	.51**	–								
IDL: Communication with friends	.61**	.56**	–							
IDL: Free time	.45**	.39**	.50**	–						
IDL: Managing time	.51**	.48**	.53**	.50**	–					
Overall IDL score	.80**	.76**	.82**	.72**	.78**	–				
QDE: Time and efficiency	.33**	.29**	.36**	.38**	.41**	.45**	–			
QDE: Well-being	.41**	.29**	.36**	.48**	.38**	.49**	.63**	–		
QDE: Social connectedness	.51**	.42**	.46**	.39**	.43**	.57**	.59**	.73**	–	
Overall QDE score	.50**	.40**	.47**	.46**	.47**	.59**	.81**	.86**	.94**	–

IDL: SR=IDL: Social Relationships; IDL: CwFam=IDL: Communication with family; IDL: CwFriends=IDL: Communication with friends; SocialConnect.=QDE: Social connectedness. *p <.05; **p <.01.

**Table 4** presents correlations between IDLS and QDES and the other Study 3 measures. Below we provide an overview of the strongest correlations for each variable. Significant correlations below *r* = .10 are not described in this section.

**Table 4:** Study 3: Intercorrelations between the Immersion in Digital Life (IDL) domains, the overall Immersion score and the subscales and overall score of Quality of Digital Experience (QDL), with the WHO-5 well-being scale, the Barratt Impulsivity subscales and sum score (BIS), the Problematic Internet Use Questionnaire (PIUQ-9), the Depression, Anxiety and Stress Scale (DASS-21), and the Satisfaction with Life Scale (SWLS).

	IDL: SR	IDL: CwFam	IDL: CwFriends	IDL: Free time	IDL: Managing time	IDL	Time& efficiency	Well-being	Social connect.	QDE
WHO-5	.04	.07*	.01	-.03	.05	.04	.14**	.18**	.15**	.17**
BIS Sum	.11**	.11**	.10**	.12**	.09*	.14**	-.10**	.00	.09*	.02
BIS: Non-planning impulsiveness	-.05	.00	-.04	.01	-.04	-.03	-.23**	-.10**	-.06	-.13**
BIS: Motor impulsiveness	.18**	.16**	.14**	.16**	.14**	.20**	.01	.09**	.19**	.13**
BIS: Attentional impulsiveness	.14**	.12**	.16**	.15**	.14**	.18**	-.01	.02	.09**	.06
PIUQ-9	.30**	.22**	.28**	.25**	.30**	.35**	.14**	.15**	.30**	.25**
DASS-21: Depression	.12**	.03	.09*	.14**	.08*	.12**	-.03	.00	.05	.02
DASS-21: Anxiety	.21**	.15**	.18**	.16**	.18**	.23**	.03	.04	.18**	.12**
DASS-21: Stress	.18**	.09*	.17**	.17**	.17**	.20**	.04	.02	.13**	.09**
SWLS	.01	.08*	.01	-.06	.05	.02	.14**	.11**	.13**	.14**

IDL: SR=IDL: Social Relationships; IDL: CwFam=IDL: Communication with family; IDL: CwFriends=IDL: Communication with friends; SocialConnect.=QDE: Social connectedness. *p <.05; **p <.01.

As expected, IDLS indicated significant positive correlations with problematic internet use, anxiety, stress, depression and impulsiveness. All the subscales and the overall score of QDES were positively connected with well-being, satisfaction with life and problematic internet use. Some specific associations were found for QDES dimensions.

## 6 Discussion

The main objective of the presented studies was to develop and validate new measures of digital behavior and experiences of digital life. The analysis presented resulted in the development of the *Quality of Digital Experience Scale* and the *Immersion in Digital Life Scale*, which can be used together or separately (see Appendix A in the **Supplementary Material** for the final versions). The analyses indicated good psychometric properties, and the scales demonstrated excellent reliability and associations with other psychological constructs.

### 6.1 Scale summary

The factor analysis indicated that the QDES consists of three factors: 1) *Well-being*; 2) *Social Connectedness*; 3) *Time & Efficiency*. Quality of Digital Experience is understood as individuals’ perceptions of their overall lived experience of DT usage and its impact on core aspects of life. The *Well-being* subscale is defined as the extent to which DT affects mental health, mood, relaxation, and overall enjoyment of life. The construct refers to the impact of DTs on psychological functioning. The *Social Connectedness* subscale is defined as the extent to which DT supports social relationships, fostering a sense of community, belonging, and inclusion. This construct measures the impact of DTs on social relationships. The *Time and Efficiency* subscale is defined as the extent to which DT promotes efficiency and facilitates the performance of everyday activities. This construct measures the effects of DTs on the efficiency, flexibility, speed, and ease of tasks performed during everyday life. The factor analysis supported a one-factor model for the IDLS, but single questions can be used as indicators of the pervasiveness of DT use in specific domains of life as well. IDL is defined as the extent to which DT is used in everyday social relationships, communication with friends and family, managing daily tasks and during free time.

The subscales of the QDES align with Seligman’s (22) conceptualization of well-being which includes the fostering of positive emotions, engagement, relationships, meaning, and accomplishment. Specifically, the well-being subscale aligns with Seligman’s conceptualization of positive emotions. The social connectedness subscale aligns with Seligman’s constructs of engagement, relationships and meaning and the time and efficiency subscale relates to accomplishment. In line with Seligman (22), we believe that focusing digital activities which promote or enhance these factors may enhance overall wellbeing. Critically, this suggests that wellbeing in relation to DT is not solely based on the mitigation or absence of negative emotional outcomes (i.e. stress and anxiety) but also on the development and maintenance of positive emotional states.

The QDES and IDLS advance our ability to measure the use and experience of digital media across multiple facets of life. Unlike many existing measures, they are not specific to individual devices, circumstances (work vs. home) or platforms, and are designed for use with the general population rather than those experiencing problematic behaviors or for use in a specific context (e.g. professional). They therefore offer researchers the opportunity to study the totality of DT use on behavior and experiences, rather than focusing solely on a narrow range of specific digital activities. In a world in which the rate of DT development far exceeds the pace at which measures of digital impact are developed, and digital devices are becoming ever more embedded in every aspect of life, the universal nature of our measures is critical to understanding the true impact of DT on people now and in the future.

### 6.2 Relations to other measures

The validation suggests that immersion in digital life and quality of digital experience have positive and negative associations with other psychological constructs. Individuals who use more DT appear to experience greater connectedness, productivity and empowerment in the context of their mobile phone use and at the same time might experience feelings of depression, anxiety, stress, and technology addiction. Comparable findings were observed for the quality of digital experience. As expected, quality of digital experience was associated with feelings of connectedness, empowerment and productivity in relationship with the mobile phone, life satisfaction and well-being. However, in contrast to our predictions, quality of digital experience and its dimensions overlapped with the concepts of addiction and anxious attachment to the mobile phone and a dependency on technology and problematic internet use. There were also some connections between Social Connectedness (QDES) and the overall QDES score with anxiety and stress. Collectively, these findings suggest that our use and experience of DT have positive and negative associations.

Whilst at first sight it may seem to be paradoxical, research often observes that digital media use evokes both benefits and harms (48). A cross-sectional analysis of the benefits and harms of UK internet use shows that benefits and harms are strongly positively related to each other (49). Benefits included more behavioral and situational factors related to economic, social and health benefits. Harms included cybersecurity concerns such as receiving a virus, a misrepresented product, a request for bank details, receiving spam, accidently encountering a pornographic website, and having credit card details stolen. Critically, both benefits and harms were strongly positively related to each other, suggesting it may not be possible to have one without the other (49, 50).

The benefits and harms of digital media use observed in this study are in line with the Mobile Connectivity Paradox (50), which describes tensions resulting from everyday connectivity. On the one hand, DT facilitates autonomy and allows users to stay in touch with family and friends independently of time and space limits, and be entertained anywhere, resulting in positive experiences (51) and greater connectedness (52). However, on the other hand, mobile connectivity may induce symptoms of stress and depression (53) because of social expectations about permanent availability (54) and the difficulty in deciding when to connect and when to disconnect (50).

Although the correlations observed do not allow us to draw conclusions about causation, the associations between greater immersion in digital life and quality of digital experience, and greater anxiety and stress may be explained by the social compensation hypothesis (55, 56). Here, the “poor get richer” hypothesis refers to individuals who struggle with anxiety, social anxiety or depression experiencing greater well-being, exploring more social relationships and improving their social skills as a consequence of using the internet and engaging in online self-disclosure (e.g. 57, 58). Such findings are supported by research showing that being active on Twitter/X, and having a larger social network, supports individuals with depressive symptoms who do not receive social support in the offline world (59).

Enhancing digital wellbeing therefore requires an approach which not only aims to reduce exposure to negative content and subsequent negative emotions, but also a positive psychology approach which aims to increase wellbeing by promoting relatedness, connectedness, positive meaning and accomplishment through DT use. Exercises derived from positive psychology, for example, workshops and exercises which aim to promote resilience, gratitude and optimism and enhance goal setting (60) may help individuals to manage the challenges and maximize the benefits of DT immersion.

The relationships between personality traits and digital immersion and engagement suggest that basic individual traits are important for our relationship with the digital world. Immersion correlated negatively with conscientiousness, meaning that higher immersion in the digital world was associated with lower conscientiousness. This finding replicates previous work showing that less conscientious individuals use the Internet more (61), tend to excessively engage in online gaming (62) and send more texts (63). However, it is noteworthy that other work has failed to observe a relationship between conscientiousness and excessive Internet use (64). Without knowing the causal direction of this relationship, we may hypothesize that either less conscientious individuals tend to be more digitalized or that more digitalized individuals, because of being immersed in the attractive digital world, tend to stay in the online world and are possibly less reliable.

The consistent (yet small) correlations between the IDLS and QDES with impulsivity further demonstrate the complex interactions between DT use and personality traits. In line with our predictions, greater levels of digital immersion were associated with greater trait impulsiveness. Contrary to expectations however, quality of digital experience also increased with greater trait-impulsiveness. Impulsivity is conceptualized as a behavioral pattern, in which future negative consequences have limited influence on the planning of actions (65). Our results therefore suggest that it is not only the extent of immersion in DT but also positive digital experiences that correlate with impulsivity. Critically, the correlation between impulsive tendencies and quality of digital life indicates that there are rewarding consequences of DT among impulsive individuals, and that greater impulsivity may contribute to greater immersion to obtain these rewards. Only the subscale of non-planning impulsivity shows (small) detrimental effects on DT experience: the greater non-planning impulsivity the lower positive digital experiences in the area of Time and efficiency and Well-being.

## 7 Limitations and future directions

Despite making a significant contribution to our ability to holistically measure DT engagement and experience, the presented studies have some limitations. Firstly, the use of cross-sectional correlational analysis does not allow for conclusions concerning causality which limits our ability to understand the mechanisms that moderate or mediate the relationships between DT and health and well-being.

Secondly, we did not test-retest the final version of the questionnaires meaning that we are unsure how stable these constructs are over time. Future research should therefore focus on longitudinal studies exploring the relationships of immersion in digital life and quality of digital experiences with other constructs and in particular, the associations with negative outcomes. Longitudinal analysis would clarify whether immersion in digital life and quality of digital experience are stable characteristics or fluctuate over time and may inform us about the trajectory of the relationship between DT use and health and well-being. We hypothesize that both measured constructs may change over time depending on situation, circumstances, life events or current psychological state.

Thirdly, for brevity, the analysis of age, gender and level of education were not considered in the current manuscript. Future detailed analyses are therefore required to understand how demographic factors may influence and act as barriers and facilitators to high quality digital experiences. Furthermore, although we can generalize the findings thanks to a representative sample in the UK, we did not validate these questionnaires among specific samples, for example, marginalized groups (people with intellectual or physical disabilities), minority groups, or clinical populations for whom immersion in digital life and the significance of positive digital experiences may be different. Future research should therefore seek to examine quality of digital experience in a broader range of populations.

Fourthly, the analysis of personality traits should be interpreted with caution because the conscientiousness, openness and agreeableness subscales employed in this study exhibited low reliability (*α = .*51;.57;.38). Low reliability may characterize instruments with a low number of items, such as the Big Five Inventory, which only has two items per subscale. Therefore, the results should be replicated using another personality scale.

Finally, whilst the relationships observed in this study demonstrate the importance of studying digital experiences in a non-domain, device or platform specific manner, the universal nature of the QDES and IDLS means that they do not provide specific information about digital experiences on specific platforms or devices. Emergent evidence suggests that digital experience is not monolithic, and that different digital domains (e.g., gaming, social media, work platforms) may have their own literacies and unique impacts on well-being and social connectedness (66). It is therefore possible that specific types of behaviors, literacies and competencies may moderate the impact of DT on wellbeing.

## 8 Implications

With growing societal concern about the impact of DT use on wellbeing there is a need for systematic evaluation of the effectiveness of interventions designed to reduce digital harms and promote digital wellbeing. The QDES and IDLS should therefore be used in future research which seeks to test the efficacy of new and existing interventions that aim to improve digital wellbeing. In particular, the QDES and IDLS may be particularly effective tools for quantifying and harnessing the impact of interventions aiming to increase digital and gaming literacy in educational settings.

## 9 Conclusions

Given the pervasive integration of DT into daily life and the increasing research interest in this phenomenon the presented project has a substantial societal and scientific impact. The current studies offer new conceptualizations and measures of digital immersion and quality of digital experiences. These studies contribute to positive cyberpsychology expanding its methodological tools and approaches. The presented measures may be used in future research that aims to explore technology from a wider perspective without focusing on a very specific context or device. Critically, our studies demonstrate that these tools facilitate a more comprehensive examination of technology use and experience, specifically addressing the often-overlooked positive aspects of DT use. They may therefore be valuable tools for policymakers, educators, and mental health professionals to better understand and address the effects of DT on individuals and communities.

## Data Availability

The raw data supporting the conclusions of this article will be made available by the authors, without undue reservation.

## Supplementary material

Supplementary Figure S1 The final model of the Immersion in Digital Life Scale obtained in Study 2 after the exclusion of one item.

Supplementary Figure S2 The final model of the Quality of Digital Experience Scale obtained in Study 2 after the exclusion of items no 3 and 4.

Supplementary Figure S3 The final model of the Immersion in Digital Life Scale obtained in Study 3.

Supplementary Figure S4 The final model of the Quality of Digital Experience Scale obtained in Study 3.
