# Supplementary figures and images for: New measurements of digital technology use: the Immersion in Digital Life and Quality of Digital Experience scales
Source: Front Psychiatry. 2025 Jun 30;16:1595536. doi: 10.3389/fpsyt.2025.1595536 (PMC12257311; doi:10.3389/fpsyt.2025.1595536)

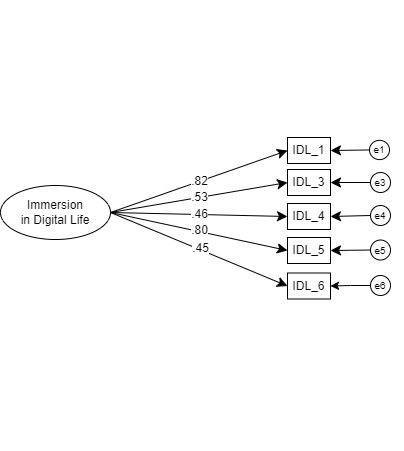

Supplement: Supplementary Figure S1 — The final model of the Immersion in Digital Life Scale obtained in Study 2 after the exclusion of one item. [file Image1.jpeg]

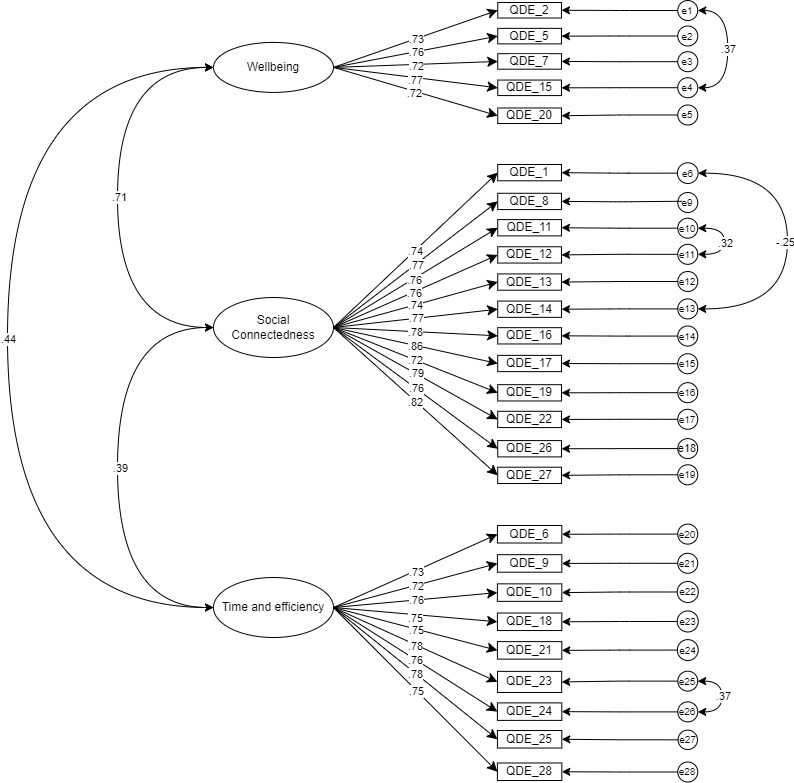

Supplement: Supplementary Figure S2 — The final model of the Quality of Digital Experience Scale obtained in Study 2 after the exclusion of items no 3 and 4. [file Image2.jpeg]

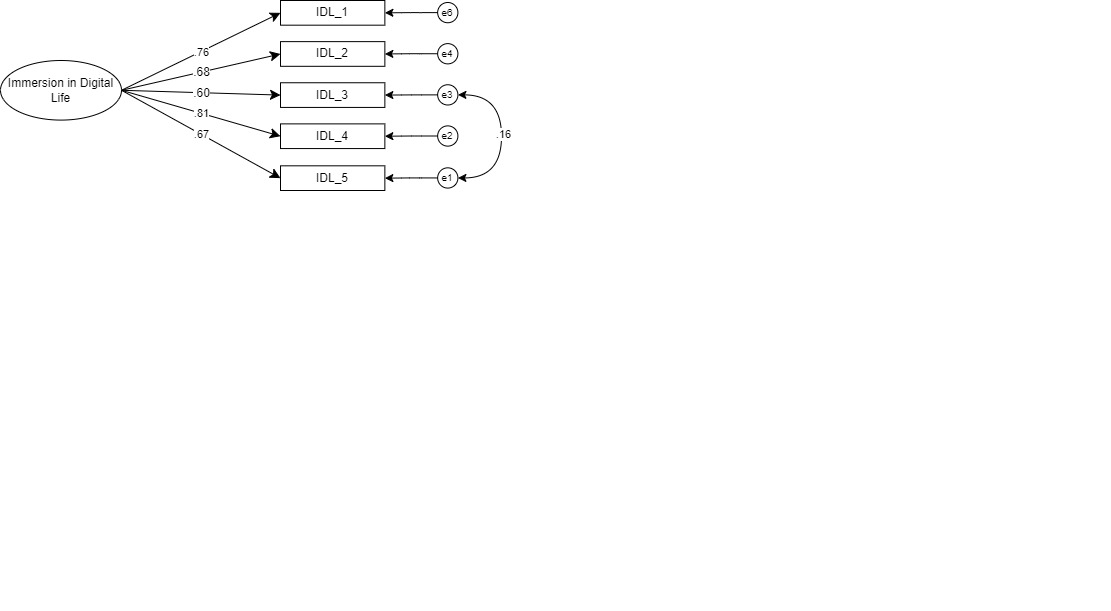

Supplement: Supplementary Figure S3 — The final model of the Immersion in Digital Life Scale obtained in Study 3. [file Image3.jpeg]

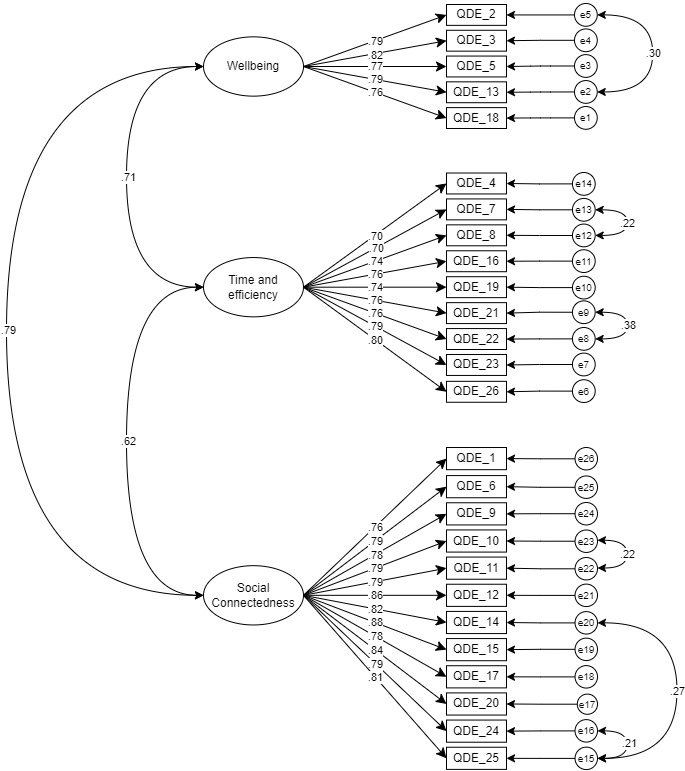

Supplement: Supplementary Figure S4 — The final model of the Quality of Digital Experience Scale obtained in Study 3. [file Image4.jpeg]
