# Supplementary material for: New measurements of digital technology use: the Immersion in Digital Life and Quality of Digital Experience scales
Source: Front Psychiatry. 2025 Jun 30;16:1595536. doi: 10.3389/fpsyt.2025.1595536 (PMC12257311; doi:10.3389/fpsyt.2025.1595536)
Supplement: Supplementary file 5 [file SupplementaryFile1.docx]

**Supplementary Materials**

**Section 1: Study 1 Supplementary Materials**

**Section 1.1: Study 1 Details of item development and selection**

The pool of items was created by six teams from six European countries based on their interviews and referring to the above-mentioned facets of DT use. Twenty-nine items, which referred to the presence of DT in everyday life, were generated and a total of 309 items in five factors which build experiences of DT use.

The first four authors of this paper who led the questionnaire development selected the pool of items to test in the study. The selection of items was based on procedures used in content validity reviews and the Delphi method (Boateng et al., 2018). The authors independently reviewed every item answering the following questions: *”*Does the item fit the subscale?” *(yes* or *no*) and “How relevant/essential is the component measured by the item for the subscale?” (*essential; useful, but not essential; not necessary)*. The reviews were compared and discussed in depth by the authors in several meetings. As a result of this process 9 items remained in the Immersion in Digital Life Scale and 136 items in the Quality of Digital Experience Scale. Subsequently, these items were rephrased (if necessary) to unify and simplify them (avoiding frequency adverbs) and maintain a clear and easy to understand structure referring to lived, personal experience.

The selected items were validated by four individuals with extensive experience in psychological research and/or the investigation of digital technology (hereafter called "experts") and three laypersons (hereafter called "non-experts") and were freely commented on by the members of the consortium teams. The experts and non-experts received a template review form with instructions, questions and factor definitions. Both experts and non-experts reviewed the items according to their *relevance:* “How essential is the item for measuring the construct described in the definition?” and *clarity*: “Is it clear what the item refers to?” and could add comments. Moreover, experts were asked about *item fit*: “Is the item a good fit for the subscale definition?” and non-experts about *difficulty*: “How difficult would it be to answer this question?” For *relevance*, the content validity ratio (CVR) was calculated (Lawshe, 1975). The items with a CVR below 0.5 were removed. The rest of the reviews were discussed in detail. Following the evaluation, seven items of Immersion and 95 items of the Quality of Digital Experience part were selected to examine the factorial structure.

**Section 1.2: Study 1 Demographic details of study 1 participants**

Table 1s: *Demographic details of study 1 participants (N = 925)*

| Age in years |  |
| --- | --- |
| Mean | 41.25 |
| SD | 13.76 |
| Range | 18-78 |
|  |  |
| Gender |  |
| Male | 440 (47.57%) |
| Female | 474 (51.24%) |
| Transgender | 3(0.32%) |
| Non-binary/third gender | 5 (0.54%) |
| Other | 1 (0.11%) |
| Prefer not to say | 2 (0.22%) |
|  |  |
| Education |  |
| Primary school | 3 (0.32%) |
| Secondary school (such as GCSE) | 95(10.27%) |
| Secondary school or college (such as A levels) | 152 (16.43%) |
| Vocational, trade or technical education | 102 (11.03%) |
| Bachelor’s degree | 389 (42.05%) |
| Master’s degree | 127 (13.73%) |
| Doctorate degree | 31 (3.35%) |
| Professional degree | 18 (1.95%) |
| Other | 5 (0.54%) |
| Prefer not to say | 3 (0.32%) |

**Section 2: Study 2 Supplementary Materials**

**Section 2.1 Demographic details of study 2 participants**

Table 2s: *Demographic details of study 2 participants (N = 474)*

| Age in years |  |
| --- | --- |
| Mean | 42.49 |
| SD | 14.88 |
| Range | 18-78 |
|  |  |
| Gender |  |
| Male | 213 (44.94%) |
| Female | 253 (53.38%) |
| Transgender | 4 (0.84%) |
| Non-binary/third gender | 1 (0.21%) |
| Other | 0 (0%) |
| Prefer not to say | 3 (0.63%) |
|  |  |
| Education |  |
| Primary school | 0 (0%) |
| Secondary school (such as GCSE) | 56 (11.81%) |
| Secondary school or college (such as A levels) | 85 (17.93%) |
| Vocational, trade or technical education | 42 (8.86%) |
| Bachelor’s degree | 202 (42.62%) |
| Master’s degree | 64 (13.50%) |
| Doctorate degree | 10 (2.11%) |
| Professional degree | 9 (1.90%) |
| Other | 3 (0.63%) |
| Prefer not to say | 3 (0.63%) |

**Section 2.2: Study 2 materials**

As in study 1, participants answered questions about their gender, age and highest level of education, the *Immersion in Digital Life Scale* (6 items) and *Quality of Digital Experience Scale* (28 items).

*The Media and Technology Usage and Attitudes Scale* (MTUAS; Rosen et al., 2013) measured the use of and attitudes towards DT. The first part of the MTUAS assesses the frequency of different digital behaviors carried out on various devices. 7 subscales from the *Media and Technology* *Usage* questionnaire were used in this study: Smartphone Usage (9 items), General social media usage (9 items), Internet searching (4 items), Media sharing (4 items), Text messaging (3 items), Phone calling (2 items) and TV viewing (2 items). The General social media usage subscale, which focuses on enquiring about the frequency of respondents’ use of Facebook, was adapted by replacing the word “Facebook” by “social media” in its items and was only presented to participants with a social media account. Answers were provided on a 10-point scale ranging from 1 – *never* to 10 – *all the time*. The second part of the MTUAS, which measures “attitudes toward technology”, was employed in full. It comprised 16 items across four subscales (Positive Attitudes Toward Technology (6 items), Anxiety About Being Without Technology/Dependence on Technology (3 items), Negative Attitudes Toward Technology (3 items) and Preference for Task Switching (4 items)). Respondents are required to indicate their level of agreement on a 5-point Likert scale ranging from 1 – *strongly disagree* to 5 - *strongly agree*.

The *Mobile Phone Affinity Scale* (MPAS; Bock et al., 2016) was employed as a second measure of DT use. The 24-item questionnaire is made up of six subscales (Connectedness, Productivity, Empowerment, Anxious Attachment, Addiction, and Continuous Use) each containing four items designed to capture positive, negative or neutral aspects of respondents’ relationship with their mobile phone. Participants responded using a 5-point Likert-scale ranging from *not at all true* to *extremely true*.

The *Satisfaction with Life Scale* (SWLS) (Diener et al., 1985) measured cognitive judgements of contentment with life. The SWLS has 5 items answered with 7-point Likert scales (*strongly disagree* – *strongly agree*). Item scores were summed, with a greater score indicating greater satisfaction with life.

*The Big Five Inventory* (BFI-10; Rammstedt and John, 2007) measured personality by assessing levels of five traits: extraversion, agreeableness, conscientiousness, neuroticism, and openness. The instrument prompts respondents to indicate how well 10 statements beginning with “I see myself as someone who…” capture their personality. Responses were given using a 5-point Likert response scale ranging from *disagree strongly* to *agree strongly*.

**Section 2.3 Descriptive statistics and reliabilities for all the variables included in Study 2**

Table 3s

*Descriptive statistics and reliabilities for all the variables included in Study 2*

| Variables | Study 2 | | |
| --- | --- | --- | --- |
|  | *M* | *SD* | *α* |
| MTUAS: Text messaging | 6.13 | 1.79 | .64 |
| MTUAS: Smartphone usage | 5.63 | 1.50 | .89 |
| MTUAS General social media usage | 4.46 | 1.55 | .89 |
| MTUAS: Internet searching | 5.85 | 1.53 | .77 |
| MTUAS: Media sharing | 3.13 | 1.70 | .82 |
| MTUAS: Phone calling | 4.72 | 1.89 | .71 |
| MTUAS: TV watching | 4.41 | 1.97 | .60 |
| MTUAS: MTU questionnaire | 4.94 | 1.14 | .92 |
| MTUAS: Positive attitude | 3.78 | 0.61 | .78 |
| MTUAS: Negative attitude | 3.25 | 0.89 | .77 |
| MTUAS: Anxiety/  dependency | 3.32 | 0.96 | .81 |
| MTUAS: Task switching | 2.75 | 0.97 | .92 |
| MPAS: Connectedness | 3.24 | 0.93 | .81 |
| MPAS: Productivity | 2.83 | 1.19 | .89 |
| MPAS: Empowerment | 2.98 | 0.97 | .86 |
| MPAS: Anxious attachment | 2.74 | 1.15 | .92 |
| MPAS: Addiction | 2.62 | 1.08 | .87 |
| MPAS: Continuous use | 2.53 | 0.99 | .76 |
| MPAS | 2.82 | 0.87 | .95 |
| Satisfaction with life | 20.90 | 7.23 | .93 |
| Extraversion | 5.63 | 1.78 | .80 |
| Agreeableness | 6.95 | 1.73 | .38 |
| Conscientiousness | 7.43 | 1.72 | .51 |
| Neuroticism | 5.95 | 2.20 | .70 |
| Openness | 7.03 | 1.94 | .57 |

*Note*. MPAS = The Mobile Phone Affinity Scale; MTUAS = The Media and Technology Usage and Attitudes Scale

**Section 2.4 Descriptive statistics for Immersion in Digital Life and Quality of Digital Experience in Study 2 and Study 3**

Table 4s*: Descriptive statistics for Immersion in Digital Life and Quality of Digital Experience in Study 2 and Study 3*

| Variables | Study 2 | | Study 3 | |
| --- | --- | --- | --- | --- |
|  | *M* | *SD* | *M* | *SD* |
| Immersion in Digital Life | 53.56 | 17.05 | 52.19 | 19.51 |
| Time and efficiency | 3.90 | 0.64 | 3.86 | 0.64 |
| Health and well-being | 3.44 | 0.76 | 3.48 | 0.81 |
| Social connectedness | 3.28 | 0.77 | 3.32 | 0.86 |
| Quality of Digital  Experience | 3.52 | 0.59 | 3.54 | 0.68 |

**Section 2.5: Spearman-Brown, Guttman and correlation coefficient obtained in Study 2 and Study 3**

Table 5s: *Spearman-Brown, Guttman and correlation coefficient obtained in Study 2 and Study 3*

|  | Study 2 | | | Study 3 | | |
| --- | --- | --- | --- | --- | --- | --- |
|  | Correlation between forms | Spearman-Brown coefficient | Guttmann split-half  coefficient | Correlation between forms | Spearman-Brown coefficient | Guttmann split-half  coefficient |
| Immersion in Digital Life | .61 | .77 | .75 | .76 | .87 | .84 |
| Time and efficiency | .88 | .94 | .92 | .87 | .93 | .92 |
| Well-being | .76 | .87 | .85 | .84 | .91 | .88 |
| Social connectedness | .91 | .95 | .95 | .94 | .97 | .97 |
| Quality of Digital  Experience | .93 | .97 | .97 | .96 | .98 | .98 |

**Section 3: Study 3 Supplementary Materials**

**Section 3.1 Demographic details of study 3 participants**

Table 6s

*Demographic details of study 3 participants (N = 830)*

| Age in years |  |
| --- | --- |
| Mean | 45.26 |
| SD | 13.30 |
| Range | 18-73 |
| Gender |  |
| Male | 402 (48.43%) |
| Female | 424 (51.08%) |
| Transgender | 1 (0.12%) |
| Non-binary/third gender | 3 (0.36%) |
| Other | 0 (0 %) |
| Prefer not to say | 0 (0 %) |
| Education |  |
| Primary school | 1 (0.12%) |
| Secondary school (such as GCSE) | 201 (24.22%) |
| Secondary school or college (such as A levels) | 154 (18.55%) |
| Vocational, trade or technical education | 149 (17.95%) |
| Bachelor’s degree | 217 (26.14%) |
| Master’s degree | 75 (9.04%) |
| Doctorate degree | 7 (0.84%) |
| Professional degree | 23 (2.77%) |
| Other | 3 (0.36%) |
| Prefer not to say | 0 (0%) |

**Section 3.2: Study 3 materials**

Demographic information was collected as in Studies 1 and 2.

The five-item version of the *Immersion in Digital Life Scale* was used. The 26-item version of the *Quality of Digital Experience Scale* was tested. For a description of the scales see Section 3.2.3. Materials.

*The Wellbeing Index* (WHO-5; Topp et al., 2015) measured subjective wellbeing. The 5-item questionnaire contains statements designed to assess the perceived amount of time over the past two weeks during which respondents experienced positive mood, relaxation, vitality, good rest and interest in their daily life. Responses are provided on a 6-point Likert scale ranging from 5 - *all the time* to 0 - *at no time.*

Trait impulsiveness was assessed by the *Barratt Impulsiveness Scale 11* (BIS-11; Patton et al., 1995), a 30-item questionnaire allowing for the measurement of different aspects of impulsiveness. Scores were computed for three subscales (Attentional Impulsiveness, Motor Impulsiveness and Non-Planning Impulsiveness). A 4-point Likert scale with the anchors of 1 - *rarely/never* and 4 - *almost always/always* was used.

The 9-item *Problematic Internet Use Questionnaire* (PIUQ-9; Koronczai et al., 2011), a measure assessing problematic internet use was included in our study. This instrument contains nine questions enquiring about the frequency with which respondents experience signs of problematic engagement with the internet, such as the use of the internet despite a preference for sleep or a desire to reduce internet use in combination with a lack of success in doing so. Responses are selected from a 5-point Likert scale ranging from 1 - *never* to 5 - *always/almost always*, with greater numbers signifying an increased risk of problematic internet use.

To assess symptoms of common mental health problems, the 21-item *Depression, Anxiety and Stress Scale* (DASS-21) (Henry and Crawford, 2005; Lovibond and Lovibond, 1995) was administered. The instrument is composed of three subscales of 7 items each, which measure levels of depression, anxiety and stress, respectively. Respondents are required to indicate the extent to which each statement applied to them “over the past week” by selecting a response on a 4-point Likert scale ranging from 0 (*did not apply to me at all*) to 3 (*applied to me very much or most of the time*). The DASS-21 has been widely used and was validated in a non-clinical UK sample, for which a Cronbach’s alpha of 0.93 was reported (Henry and Crawford, 2005).

*The Satisfaction with Life Scale* (SWLS) used in study 2 was employed in this study as well.

References

Boateng, G. O., Neilands, T. B., Frongillo, E. A., Melgar-Quiñonez, H. R., Young, S. L. (2018). Best practices for developing and validating scales for health, social, and behavioral research: A primer. *Front. Public Health* 6, Article 149. <https://doi.org/10.3389/fpubh.2018.00149>

Bock, B. C., Lantini, R., Thind, H., Walaska, K., Rosen, R. K., Fava, J. L. et al. (2016). The Mobile Phone Affinity Scale: Enhancement and refinement. *JMIR Mhealth Uhealth* 4, e134. <https://doi.org/10.2196/mhealth.6705>

Diener, E., Emmons, R. A., Larsen, R. J., Griffin, S. (1985). The satisfaction with life scale. *J. Pers. Assess.* 49, 71–75. <https://doi.org/10.1207/s15327752jpa4901_13>

Henry, J. D., Crawford, J. R. (2005). The short-form version of the Depression Anxiety Stress Scales (DASS-21): Construct validity and normative data in a large non-clinical sample. *Br. J. Clin. Psychol.* 44, 227–239. <https://doi.org/10.1348/014466505X29657>

Koronczai, B., Urbán, R., Kökönyei, G., Paksi, B., Papp, K., Kun, B. et al. (2011). Confirmation of the three-factor model of problematic internet use on off-line adolescent and adult samples. *Cyberpsychol. Behav. Soc. Netw.* 14, 657–664. <https://doi.org/10.1089/cyber.2010.0345>

Lawshe, C. H. (1975). A quantitative approach to content validity. *Pers. Psychol.* 28, 563–575.

<https://doi.org/10.1111/j.1744-6570.1975.tb01393.x>

Lovibond, P. F., Lovibond, S. H. (1995). The structure of negative emotional states: Comparison of the Depression Anxiety Stress Scales (DASS) with the Beck Depression and Anxiety Inventories. *Behav. Res. Ther.* 33, 335–343. <https://doi.org/10.1016/0005-7967(94)00075-U>

Patton, J. H., Stanford, M. S., Barratt, E. S. (1995). Factor structure of the Barratt impulsiveness scale. *J. Clin. Psychol.* 51, 768–774.

Rammstedt, B., John, O. P. (2007). Measuring personality in one minute or less: A 10-item short version of the Big Five Inventory in English and German. *J. Res. Pers.* 41, 203–212. <https://doi.org/10.1016/j.jrp.2006.02.001>

Rosen, L. D., Whaling, K., Carrier, L. M., Cheever, N. A., Rokkum, J. (2013). The Media and Technology Usage and Attitudes Scale: An empirical investigation. *Comput. Hum. Behav.* 29, 2501–2511. <https://doi.org/10.1016/j.chb.2013.06.006>

Topp, C. W., Østergaard, S. D., Søndergaard, S., Bech, P. (2015). The WHO-5 Well-Being Index: A systematic review of the literature. *Psychother. Psychosom.* 84, 167–176. <https://doi.org/10.1159/000376585>

**Section 3.3 Descriptive statistics and reliability study 3**

Table 7s

*Descriptive statistics and reliabilities for all the variables included in Study 3*

|  | Study 3 | | |
| --- | --- | --- | --- |
|  | *M* | *SD* | *α* |
| WHO-5 | 13.13 | 5.58 | .90 |
| BIS | 63.00 | 11.26 | .85 |
| BIS: Non-planning impulsiveness | 25.03 | 5.04 | .72 |
| BIS: Motor impulsiveness | 21.94 | 4.75 | .73 |
| BIS: Attentional impulsiveness | 16.03 | 3.99 | .71 |
| PIUQ-9 | 21.15 | 7.67 | .90 |
| DASS-21: Depression | 6.38 | 5.90 | .94 |
| DASS-21: Anxiety | 4.34 | 4.63 | .88 |
| DASS-21: Stress | 6.46 | 5.29 | .92 |
| SWLS | 19.51 | 7.57 | .92 |

*Note*. BIS = Barratt Impulsiveness Scale; PIUQ = Problematic Internet Use Questionnaire; SWLS = Satisfaction with Life Scale

**Appendix A**

Immersion in Digital Life Scale

The following questions are about the extent to which you use digital technology in your life. For the purposes of this questionnaire, we are defining digital technology as **devices, systems, services, data or processes that use digital information in some way, for example, computers, tablets, mobile phones or smart TVs, among other devices**. These technologies may be used for various purposes such as communication, entertainment, work, education and everyday tasks.

Read each of the questions and respond as honestly as you can thinking about your personal experience. Please drag the slider below each question to indicate **how digital** each life activity is for you. There are no good or bad answers, we want to know your personal experience.

1. To what extent are your social relationships conducted digitally?

Not at all digital Completely digital

___________________________________________________________________

1. To what extent is your communication with family digital?

Not at all digital Completely digital

___________________________________________________________________

1. To what extent are the activities you do in your free time digital?

Not at all digital Completely digital

___________________________________________________________________

1. To what extent is your communication with friends digital?

Not at all digital Completely digital

___________________________________________________________________

1. To what extent is managing your time digital?

Not at all digital Completely digital

___________________________________________________________________

**Scoring**

Total score - mean.

Single questions values.

**Quality of Digital Experience Scale**

The following questions are about your lived experience of digital technology.

For the purposes of this questionnaire, we are defining digital technology as devices, systems, services, data or processes that use digital information in some way, for example, computers, tablets, mobile phones or smart TVs, among other devices. These technologies may be used for various purposes such as communication, entertainment, work, education and everyday tasks.

Read each statement and indicate **to what extent you agree with it** by selecting **one answer** from the options provided ranging from "strongly disagree" to "strongly agree".

Please respond as honestly as you can thinking about your personal experience. There are no good or bad answers, we want to know your personal experience.

|  | 1. Strongly disagree | 2. Disagree | 3. Neither agree or disagree | 4. Agree | 5. Strongly agree |
| --- | --- | --- | --- | --- | --- |
| 1. Using digital technology helps me to deepen relationships with others |  |  |  |  |  |
| 2. Using digital technology enhances my mental wellbeing |  |  |  |  |  |
| 3. I am able to improve my mood using digital technology. |  |  |  |  |  |
| 4. Using digital technology makes it easy to complete tasks in my life |  |  |  |  |  |
| 5. Using digital technology makes my life enjoyable |  |  |  |  |  |
| 6. Using digital technology makes me feel closer to people |  |  |  |  |  |
| 7. Using digital technology enables me to do more with less effort |  |  |  |  |  |
| 8. By using digital technology I save time on everyday tasks |  |  |  |  |  |
| 9. Using digital technology makes me feel supported by other people |  |  |  |  |  |
| 10. Using digital technology helps me feel part of other people's lives |  |  |  |  |  |
| 11. Using digital technology makes me feel more connected to other people |  |  |  |  |  |
| 12. Using digital technology helps me to bond with others |  |  |  |  |  |
| 13. Using digital technology helps me to take care of my mental wellbeing |  |  |  |  |  |
| 14. Digital technology use increases my sense of belonging to a group |  |  |  |  |  |
| 15. Using digital technology strengthens my attachment to others |  |  |  |  |  |
| 16. Using digital technology saves my time |  |  |  |  |  |
| 17. Using digital technology makes my social life more fulfilling |  |  |  |  |  |
| 18. Using digital technology helps me to relax |  |  |  |  |  |
| 19. I can meet my needs more quickly using digital technology |  |  |  |  |  |
| 20. I feel closer to people like me because I use digital technology |  |  |  |  |  |
| 21. I complete tasks more quickly when using digital technology |  |  |  |  |  |
| 22. I achieve more in less time when I use digital technology |  |  |  |  |  |
| 23. Using digital technology helps me to complete tasks efficiently |  |  |  |  |  |
| 24. I find it easy to form deep connections with others when using digital technology |  |  |  |  |  |
| 25. I experience a greater sense of community thanks to using digital technology |  |  |  |  |  |
| 26. Using digital technology makes my life easier |  |  |  |  |  |

**Scoring**

Quality of Digital Experience total score - mean.

1. *Well-being*

Mean: 2, 3, 5 13, 18

1. *Social connectedness*

Meam: 1, 6, 9, 10, 11, 12, 14, 15, 17, 20, 24, 25

1. *Time & efficiency*

Mean: 4, 7, 8, 16, 19, 21, 22, 23, 26
